# Henri Matisse (1869–1954). Icarus (from the illustrated book, Jazz, published in 1947 by E. Tériade)

**DOI:** 10.3201/eid0905.AC0905

**Published:** 2003-05

**Authors:** Polyxeni Potter

**Affiliations:** * Centers for Disease Control and Prevention, Atlanta, Georgia, USA

**Figure:**
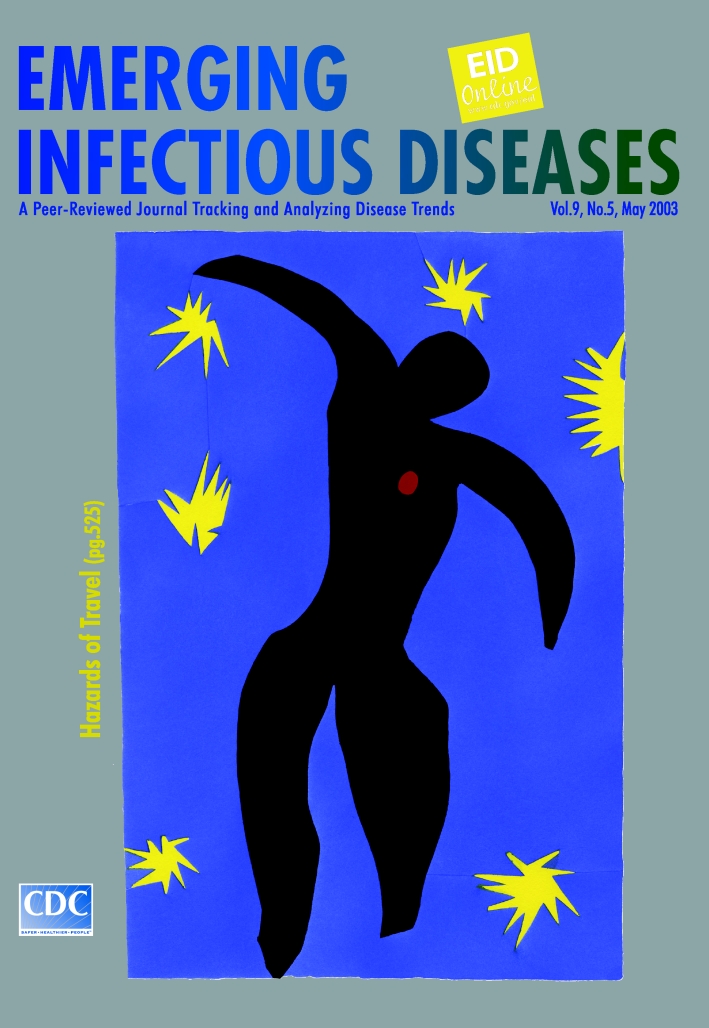
Henri Matisse (1869–1954). Icarus (from the illustrated book, Jazz, published in 1947 by E. Tériade). Copyright 2003 Succession H. Matisse, Paris / Artists Right Society, New York Reproduction, including downloading, of Matisse works is prohibited by copyright laws and international conventions without the express written permission of Artists Rights Society (ARS), New York.

“…send me a white cane,” Henri Matisse exhorted his assistants when he completed the compositions for his illustrated book Jazz. The artist was nearly blinded by working with intense color under the brilliant Mediterranean light of the south of France (*1*). To protect against glare, he used overstated hues and intense blacks. To overcome incapacitating illness, he invented a new medium, “drawing with scissors.” Cutting shapes from prepainted paper, he formed the contour and the internal area of a shape simultaneously, eliminating as he put it, “the eternal conflict between drawing and color” (*2*). These cutouts, begun as compensation for exhausting illness and confining surgery, became another creative peak near the end of the artist’s life.

Matisse started to paint while convalescing from appendicitis at age 20. He became so captivated by the joy of creative expression that within a year he abandoned his law aspirations and went to Paris to study art, in a period still reverberating with the color innovations of van Gogh, Gauguin, and Cézanne. Trained in the academic tradition by symbolist painter Gustave Moreau, Matisse used his love of the human figure and his solid footing in art history as a springboard to greatness. He became a leader of the Fauve movement, known for its radical, even violent, use of color. He broadened his artistic scope through study of Japanese prints, Persian ceramics, and Arabic designs and sought inspiration in Spain and Morocco (*3*). His long career as painter and sculptor was filled with restless experimentation, and in addition to innovative paper cutouts, his artistic efforts extended to tapestry, ceramics, stained glass, and murals. Along with Pablo Picasso, he became a pillar of 20th century art (*4*).

In Jazz, Matisse’s cutout forms are mingled with meditations on random topics, elaborately scrolled and interspersed throughout the composition. In this syncopated design (perhaps the visual counterpart of jazz music, which the artist defined as “rhythm and meaning”), figures are chromatic and rhythmic improvisations distilled to pure form (*1*). Spare and geometric, they are filled with undulating movement and circular rhythm. Even though their range is deliberately reduced, the colors are exuberant and provocative, and the harmonious compositions are filled with almost palpable light (*2*).

In “Notes of a Painter,” Matisse reflected that his goal as an artist was to uncover and record with balance and purity the “essential character” of things beneath their external appearance. “The Flight of Icarus,” on this cover of Emerging Infectious Diseases, is one of the most famous figures in Jazz. The cutout interprets the symbolic journey of Daedalus’ son (*5*) and depicts the fall of the mythologic adventurer from the azurean skies amidst “either stars or bursts of artillery fire” (perhaps reflecting the artist’s consternation in the aftermath of World War II). The pure form of the cutout, and the color that constitutes rather than clothes the form, captures the essence of human exploration.

Icarus’ stretched-out arms negotiating flight, the fiery heart cloaked in the vibrant black of its aspirations, the bright chunks of sun that proved the man’s demise freeze in a moment of exhilaration. About to end, the euphoric moment turns somber. The head is tilted away from the sun’s splendor toward the pedestrian view below. The gliding figure, closing its celestial dance and filled with exalted vertigo, is laden with the certainty of the fall.

Our age has transformed Icarian and heliotropic quests into space exploration. We orbit the globe, defying the sun and the forces of gravity, for we still long for the charged moment of discovery that comes from roaming the earth and beyond. Yet, we have conquered neither gravity nor the mundane hazards at our destinations. Like Daedalus’ crude fabrications, our wings still melt in the heat, and at travel’s end, we fall prey to biologic hazards, exotic microbes. Be it emergent viruses (such as the cause of severe acute respiratory syndrome) or common intestinal bacteria (including *Aeromonas* spp.), the most insistent plague of travelers, these hazards slow the journey and limit the height of human exploration.
